# Stratified sampling design and loss to follow-up in survival models: evaluation of efficiency and bias

**DOI:** 10.1186/1471-2288-11-99

**Published:** 2011-06-26

**Authors:** Cibele C César, Marilia S Carvalho

**Affiliations:** 1 Department of Statistics, Federal University of Minas Gerais, Belo Horizonte, Minas Gerais, Brazil; 2 National School of Public Health, Oswaldo Cruz Foundation, Rio de Janeiro, Brazil

## Abstract

**Background:**

Longitudinal studies often employ complex sample designs to optimize sample size, over-representing population groups of interest. The effect of sample design on parameter estimates is quite often ignored, particularly when fitting survival models. Another major problem in long-term cohort studies is the potential bias due to loss to follow-up.

**Methods:**

In this paper we simulated a dataset with approximately 50,000 individuals as the target population and 15,000 participants to be followed up for 40 years, both based on real cohort studies of cardiovascular diseases. Two sample strategies - simple random (our golden standard) and Stratified by professional group, with non-proportional allocation - and two loss to follow-up scenarios - non-informative censoring and losses related to the professional group - were analyzed.

**Results:**

Two modeling approaches were evaluated: weighted and non-weighted fit. Our results indicate that under the correctly specified model, ignoring the sample weights does not affect the results. However, the model ignoring the interaction of sample strata with the variable of interest and the crude estimates were highly biased.

**Conclusions:**

In epidemiological studies misspecification should always be considered, as different sources of variability, related to the individuals and not captured by the covariates, are always present. Therefore, allowance must be made for the possibility of unknown confounders and interactions with the main variable of interest in our data. It is strongly recommended always to correct by sample weights.

## Background

It is widely acknowledged, both theoretically and in practice, that incorporating design features into estimation of descriptive parameters, such as prevalence, can help avoid bias and reduce standard errors [1-4]. However, in spite of the consensus in the statistical environment, grounded in clear evidence and well established procedures to deal with complex sample strategies in survival modeling [5], these principles are quite often ignored in applied settings. For instance, recently published studies [6,7] using well-known cohort data (MESA [8] and MONICA [9]) neither incorporate design weighting into the analysis, nor discuss its appropriateness.

This paper was motivated by discussion of the sample strategy used in a recent large multi-center cohort study, with approximately 50,000 people as the target population and 15,000 participants to be followed-up for at least 20 years [10]. The participants were selected by non-proportional stratified sampling. The main aim here is to present - as clearly as possible for non-statistical researchers - the impact of ignoring sample design, and thus to contribute to improving data analysis practice in epidemiology. In this case study we evaluate the impact of sampling weights and loss to follow-up on estimation of the parameters of a Cox proportional hazard model, by evaluating bias and precision.

Stratified random sampling involves dividing the population members into non-overlapping groups called strata, defined by selected characteristics and each sampled separately. Varying sample fractions by stratum improves the efficiency of sample design and estimators for relatively small but important population subgroups. As the proportion of the samples in each stratum varies, the weight of each individual will be proportional to the inverse of the sample fraction in the respective group, as described in Kish (1965) [4]. Computing those weights gives each stratum the same relative importance as it displays in the population. In a Stratified sample, as the association between exposure and the event may vary within each stratum, estimation of the marginal association - the average association in the entire population - should consider the individual, and varying, probability of being included in the sample.

Varying sample weights across the strata may induce a difference between the probability distributions for the outcome in the sample and in the population, because of the covariates included in the model. In such cases the design carries information about the outcome, and is therefore considered informative or non-ignorable.

In a survival model, where time-to-event *T *is the response variable, **x **the covariates vector and **z **the design factor, if **z **is not related to *T *|**x**, the design factor **z **is ignorable. Boudreau and Lawless [11] analyzed the impact of sampling design on the Cox proportional hazards model, considering both clustering and stratification. If the sampling design is ignorable, both weighted and unweighted procedures are asymptotically unbiased and should yield similar point estimates. However, if the sampling design is non-ignorable, consistent estimation can be achieved by introducing design weights into the estimating functions, as proposed by Binder (1992) [12] and Lin (2000) [13].

Another major problem in long-term cohort studies is potential bias due to loss to follow-up. This problem is widely recognized and several approaches deal with it [14]. The Cox model assumes non-informative censoring.

However, this is an unwarranted assumption in long-term cohort studies, and differential losses related to the sampling strata may increase the bias. Lawless (2003) [5] discusses these issues further and considers the use of time-varying weights that deal at the same time with a non-ignorable sampling plan and non-ignorable censoring.

The next section presents the case study, describing the simulated population and two different scenarios of loss to follow-up. Next the sample plan strategies and model fitting are presented. The results section uses a graphical representation to make the discussion of the impact of ignoring sample design more accessible to non-mathematical readers.

## Methods: Simulation exercise

### The population

A population of 52750 individuals belonging to three sampling strata was generated. As the focus of our motivating exemple was a study in a working population, we defined the strata by occupational category, which relates to socioeconomic status. The groups, in descending order of occupational category, were: *professionals *(50.5%), *technicians *(28%) and *administrative *staff (21.5%). The age and socioeconomic distributions were based on an epidemiological study with census data involving all employees at a Brazilian university [15]. The prevalence for the exposure variable, *smoking*, was based on the same data: 15.9% smokers among *professionals*, 20.9% among *technicians *and 25.3% *administrative *staff. Myocardial infarction (MI) was the event of interest. To generate age at infarction, which defined time-to-event *T_i_*, we used data from a Spanish study [16]. We considered only administrative censoring at 40 years of follow-up for all surviving subjects.

**Figure 1:**
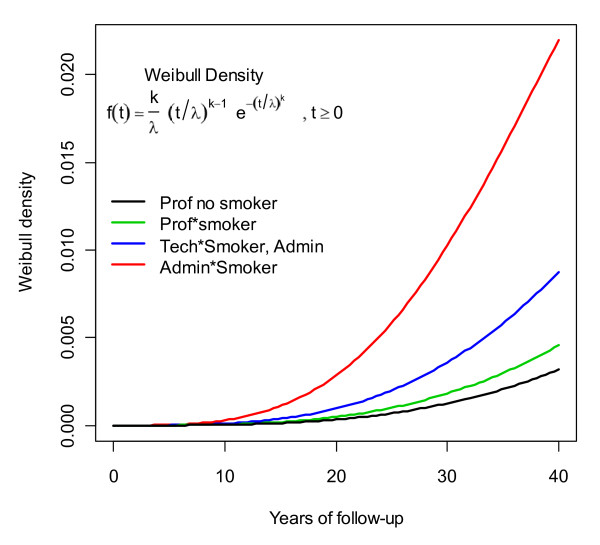
**Weibull distribution**. Effect of changing the scale parameter on the time-to-event curve based on the simulated scenario.

Smoking affected survival in interaction with the occupational position: hazard ratios of 1.5 among *professionals*, 2.0 among *technicians *and 3.0 among *administrative *staff. In addition, as the occupational strata are related to socioeconomic position, hazard increased by 50% and tripled in the *technicians *and *administrative *strata, respectively, as compared to the *professionals *stratum. Summarizing, the equation to generate the time-to-event data was:

The Weibull density equation and curves for time-to-event using the parameters above are presented in Figure 1.

### The sample plans

The sample size estimated in our motivating exemple [10] was 15,000 people. In order to increase the power in the *administrative *and *technicians *strata, these groups were oversampled: 3,000 individuals in the *professionals *group, 4,500 in the *technicians *group and 7,500 in the *administrative *staff. Therefore the weights - the inverse of the selection probability - in each group was 8.89, 3.29 and 1.50, respectively. A simple random sample was extracted for comparison.

We generated 2,000 samples, with 15,000 individuals each, for both random and stratified sample plans. To evaluate the impact of loss to follow-up we used the same samples as already simulated, censoring individuals that had experienced the event. Two different scenarios were defined: a 15% random loss and a differential loss by sample strata (*professionals *with 8% loss, *technician *with 12% and *administrative *with 20%).

### Model fitting

Each sample was fitted using Cox proportional hazards model. The first - **Full **(Eq:1) - model used the same information that generated the population, except for the parametric Weibull curve. The second - **Marginal **(Eq:2) - model included the strata as independent terms, but not interacting with our variable of interest *smoking*. The last model, without the design factor, is the **Smoke-only **(Eq:3) model.(1)(2)(3)

The population parameters for each model are given in Table 1. As we know the model and parameters that generated this population, both models 2 and 3 are incomplete. However, except in simulation studies, the complete model is never known and significant covariates are often ignored. Therefore, even under a misspecified model, it is important to compare the sample estimates with the "true" value. A Gaussian kernel was used to present the distribution of the 2,000 sample estimates for each model and strategy.

**Table 1:** Estimated Population Hazard Ratios for each fitted model

Variables	MODELS					
	Full		Marginal		Misspecified	
	HR	SE	HR	SE	HR	SE
Administrative	2.93	0.05316	3.71	0.04345	-	-
Technicians	2.55	0.05727	1.66	0.04872	-	-
Smoking	-	-	2.21	0.03758	2.47	0.0374
Prof*Smoking	1.41	0.08341	-	-	-	-
Admin*Smoking	2.93	0.05354	-	-	-	-
Tech*Smoking	1.93	0.07386	-	-	-	-

## Results and Discussion

### Comparison of sampling schemes under different models

**Figure 2:**
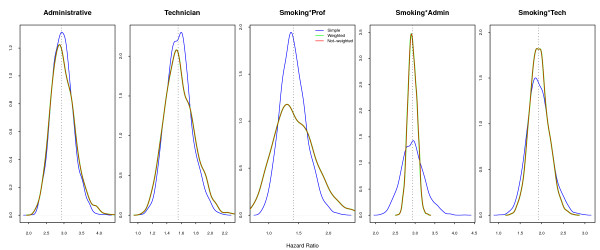
**Simulated Hazard Ratios under the Full Model**. Correctly specified model returns exactly the same results independently of considering sample weights.

Considering the **Full **model, both sample designs and fitting strategies give non-biased estimates. For the design-related variables, variance in parameter estimates is slightly smaller with simple random sampling than with weighted sampling. On the other hand, the variance in the samples for interaction of *smoking *with each professional category changes with the sample weighting: it is smaller for the *professional *stratum, larger for the *technicians *and much larger for the *administrative *group (Figure 2). Note that when the model is completely specified, whether or not the weights are included in stratified sampling, almost exactly the same point estimates are returned.

**Figure 3:**
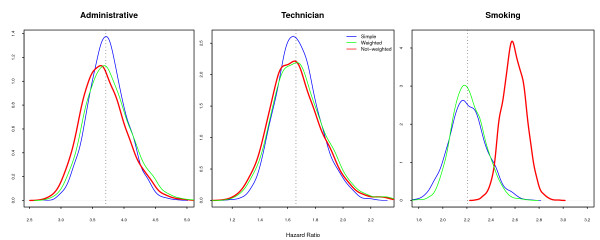
**Simulated Hazard Ratios under Marginal Model**. Large difference is observed for the hazards associated with smoking when fitting without sample weights, if the model does not include the interaction with professional category.

The **Marginal **model, with just a common effect of *smoking *across all strata, presents similar and unbiased estimates for the design factor for both sample designs (Figure 3), when compared with the **Marginal **population parameters. The hazard ratios for *smoking*, whether with random sampling or in the model with sample design correction, were similar and non-biased. However, those estimates were strongly biased when the sampling weights were not included in the model: the probability distribution for the estimates did not include the true value of the parameters, with 95% confidence. The argument in favor of not including the sample weights is that it improves precision [17,18], but in our example the increased precision excluded the true value of the parameter.

**Figure 4:**
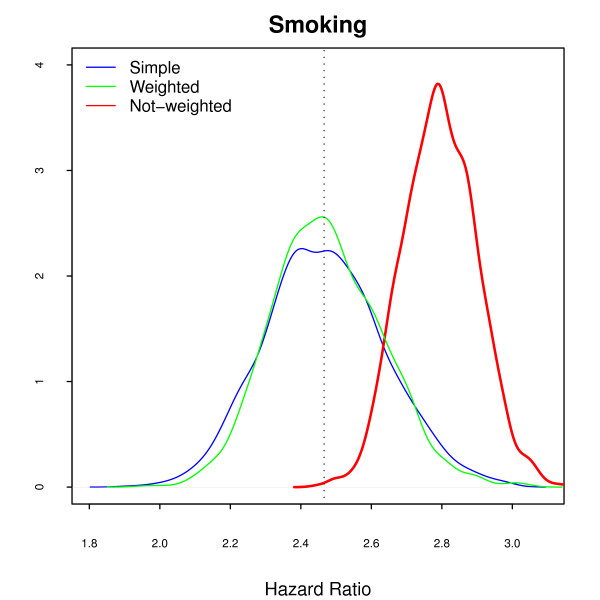
**Simulated Hazard Ratios under Smoke-only Model**. The pattern is similar to the Marginal model, with similar bias.

The **Smoke-only **model returned very similar results (Figure 4), with smaller variance but strong bias. The average risk for smoking, ignoring the interaction with professional category, is really 2.21 (Table 1). The misspecification of the model in this case caused an overestimation of the smoking effect, as it absorbed the effect of professional category. Most studies include the variable indicating the sampling strata in the model, even when ignoring the sample weights [6,7], considering that this, unfortunately insufficient, procedure will correct for the design effect. The crude estimated effect, usually used in exploratory analysis and to select the most important variables, is also misleading, as are the Kaplan-Meier estimates and Mantel-Haenszel (or log-rank) tests [19]. Although correcting for the sample weights is possible and simple, it is rarely done.

### Comparison of modeling strategies in terms of loss to follow-up

**Figure 5:**
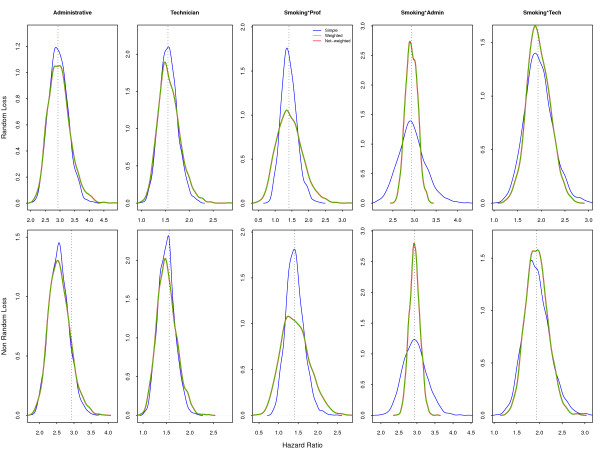
**Simulated Hazard Ratios with loss to follow-up under Full Model**. The upper frames show the random loss to follow-up and the lower ones the non-random censoring.

**Figure 6:**
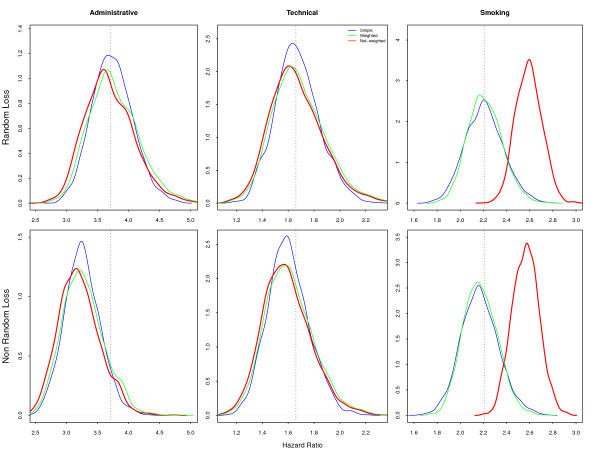
**Simulated Hazard Ratios with loss to follow-up under Marginal Model**. The upper frames, with the random loss to follow-up, show the bias for the smoking-hazard ratio for the non-weighted model. The lower frames with non-random censoring show the bias for all models.

**Figure 7:**
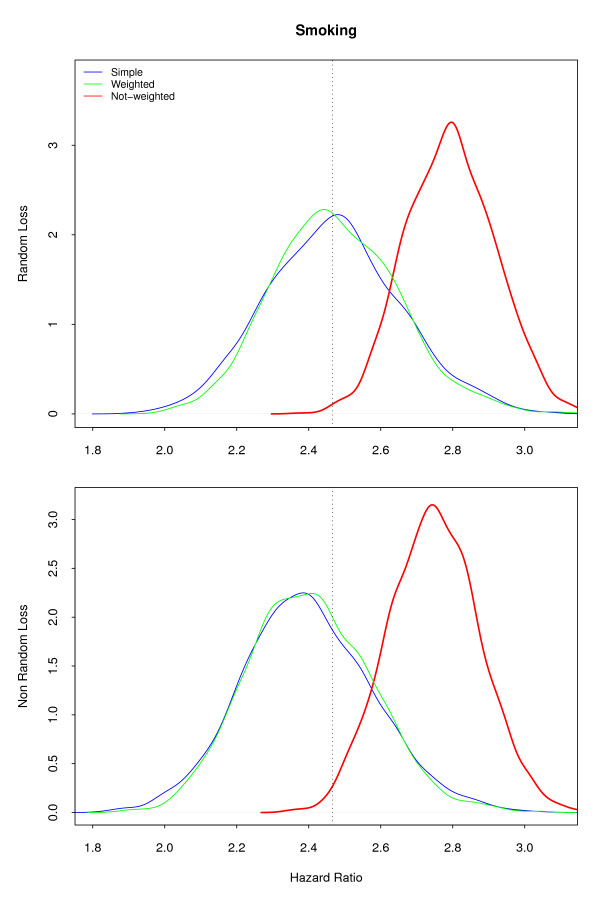
**Simulated Hazard Ratios with loss to follow-up under Smoke-only Model**. The upper frame shows the bias for the non-weighted smoke-only model and the lower one the bias for all approaches due to non-random loss.

Random loss is a non-informative censoring mechanism. Therefore it affects only precision, with results similar to those presented in the previous section (Figures 5 to 7, upper frames). If the model is well specified, the covariate associated with loss will absorb the loss to follow-up, as shown in Figure 5. As expected, because this is informative censoring, the larger losses in the *administrative *category decreases its hazard in all models and all sample strategies.

The **Marginal **model (Figure 6), with non-weighted fit, displays a bias for smoking similar to the same model without losses (Figure 3). Attrition is a recognized problem in longitudinal studies [20]. Yang and Shoptaw (2005) [21] present a thorough discussion of conceptual and practical issues in analyzing incomplete longitudinal data. However, in our simulations, the impact of ignoring the sample weights is larger than the impact of dropout, which is not as large in our example as in some of the studies discussed. The bias for smoking in the **Smoke-only **model (Figure 7) points in two directions. When the sampling weights were not included, it overestimates the hazard for smoking. On the other hand, as losses were larger in the *administrative *stratum, the values of the estimates decrease in the random sample and in the weighted model. This feature was already present, although not as visible, in the **Marginal **non-random loss to follow-up. Analyzis of the crude effect of smoking, using a Mantel-Haenszel test, should include the non-administrative censored group as a separate category.

### Overall comparison

**Figure 8:**
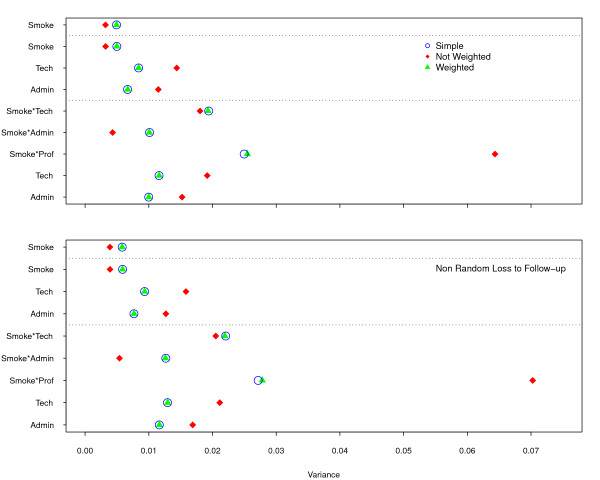
**Average Variance of Estimates according to two Scenarios: without loss and with non-random loss to follow-up**. The upper frame, without loss, shows smaller variance than the lower one and a similar pattern.

The average variance of the estimates for each covariate (Figure 8) is very similar in both weighted and random sampling models, both with and without loss to follow-up. As expected, with the smaller number of events due to the losses, the average variance shifted towards higher values. The pattern of the non-weighted model is for the mean variance for the smoking variable, isolated or in interactions, to decrease, except for smoking among professionals. The variance, in the latter case, is very large because both the total number of observations and the hazard in this category are small.

**Figure 9:**
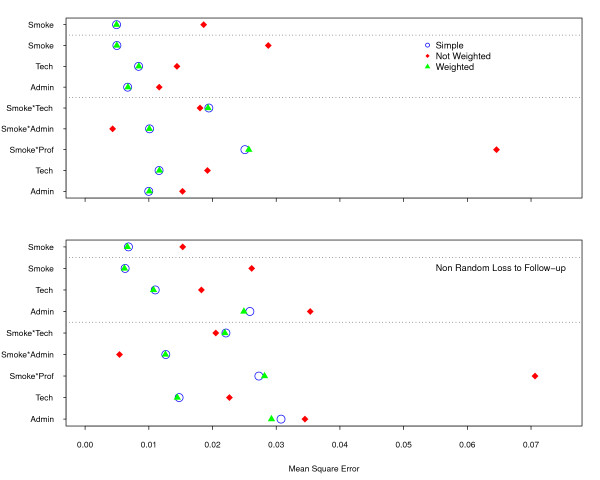
**Mean Square Error according to two Scenarios: without loss and with non-random loss to follow-up**. Both simulations, without loss (upper frame) and with loss (lower one), display a similar pattern, with the non-weighted model performing much worse.

Mean square error (MSE) is the sum of the variance and the squared bias of the estimates. This statistic is a good summary of the quality of a point estimate, as it combines the random and systematic error [22,23]. The coincidence between random sampling and weighted model in Figure 9 is the same as described previously for the average variance of the estimates. However, in the non-weighted model, the systematic error predominates, making it the worst fit for all variables, except for the interaction of *smoking *with the *technicians *and *administrative *staff. The loss to follow-up simulations displayed similar patterns, with much larger MSE.

The simulation exercise was restricted to Cox regression, with only a few scenarios. We tested many different scenarios with other covariates, omitted risk factors, and so on, but decided to present only these simpler models, so as to highlight the impact of ignoring the sample weights. Evidently, the large disparity in sample weights favored clear demonstration of the bias. However, these sample weights reflect our experience. Other modeling approaches, such as repeated measures analysis, were not implemented, and different results could be obtained.

If non-administrative censoring is considerable, then a valuable tool is to take a sub-distribution hazard approach, re-weighting individuals in the risk set. The sample weighting itself could be recalculated at each dropout [24].

## Conclusions

Quite often researchers do not include either sample weights or strata indicators in statistical models. Yeboah et al (2010) [19] used only white race in a univariate model, in spite of the four strata (white, African-American, Hispanic and Asian) that defined the sample strata in MESA [8]. Race was included as a common covariate, and excluded from the multivariate models. Neither the six study communities nor the sample weights were mentioned. Two other papers on the same cohort were more careful. Polonsky et al (2010) [25] controlled for race. Bertoni et al (2010) [6] not only included race, but tested for interaction with the main exposure variable. Neither evaluated the impact of the study communities.

Our results confirmed that, in a correctly-specified model, ignoring the weights does not change the estimated parameters, and precision may improve (a result theoretically proven for inference based on ordinary least squares) [26,27]. As suggested by Winship and Radbill (1994) [28], the decision whether or not to include the weights in the model should be based on the role of the stratifying variable. In the presence of interaction between the stratifying variable and other independent variable not included in the model, bias will be introduced if sample weights are not considered. However, the correct model is only known for simulated populations. Also strata are usually chosen to increase the sample size of populations whose characteristics are important to the outcome under study.

The primary objective of analyzing survey data is to make inferences about the population of interest [29]. Therefore survey planning starts by defining the target population, to which results will be referenced [2,30]. The role of the population of reference in analysis of survey data is related to the meaning of the error term of the statistical model. In the physical sciences, the error of a regression is considered a measurement error. Epidemiology, however, besides measurement, has to consider different sources of variability relating to individuals, and not captured by the covariates included in the model [2]. Actually, this reasoning lies behind the development of random effect ("frailty") models in survival analysis [31]. Another issue is the use of crude estimates. The usual practice in epidemiology is to control for confounders. However, public health policies may need those numbers to estimate disease burden or to evaluate the impact of targeting specific risk factors. The **Smoke-only **model (Eq:3) gives exactly the desired estimate for these purposes. The correct numbers should thus be given, using the appropriate weighting in an uncontrolled model.

The stratification by professional categories, which assigns much larger weight to the lower social stratum, was guided by the need to increase the power to detect social-related risk factors. Nevertheless, almost any covariate displays different prevalence in different socioeconomic groups. Also almost all covariates interact, positively or negatively, changing the risk. Smoking itself presents similar physiological risk across socioeconomic strata. However, belonging to the most deprived stratum implies differences in other risk factors such as larger body mass index, worse diet, inadequate exercise, all associated with cardiovascular diseases, and these are the known and easily-measured risk factors. Unknown or unreliable measures, such as stress or mental health, will always exist. Therefore allowance has to be made for the possibility of unknown confounders and interactions in our data associated with the sample strata. Rubin [32] recommends that observational studies should approximate randomized experiments, and that the assignment mechanism, in our case smoking or not smoking, should be as unconfounded as possible. Graubard and Korn (2002) [33] recommend weighted estimators, as they believe their model-free aspects outweigh their potential inefficiency. On the same reasoning, we strongly recommend always correcting by sample weights.

## Competing interests

The authors declare that they have no competing interests.

## Authors' contributions

Both authors designed, analyzed and wrote the paper.

## Pre-publication history

The pre-publication history for this paper can be accessed here:

http://www.biomedcentral.com/1471-2288/11/99/prepub
